# Supplementary material for: Linking the pressure dependence of the structure and thermal stability to α- and β-relaxations in metallic glasses
Source: Sci Adv. 2025 Oct 3;11(40):eadz7406. doi: 10.1126/sciadv.adz7406 (PMC12494008; doi:10.1126/sciadv.adz7406)
Supplement: Supplementary file 1 — Supplementary Text S1 to S5 Figs. S1 to S9 [file sciadv.adz7406_sm.pdf]

Science Advances
AAAS

Supplementary Materials for

Linking the pressure dependence of the structure and thermal stability to α- and β-relaxations in metallic glasses

Jie Shen *et al.*

Corresponding author: Jie Shen, jie.shen@neel.cnrs.fr; Beatrice Ruta, beatrice.ruta@neel.cnrs.fr

Sci. Adv. **11**, eadz7406 (2025)
DOI: 10.1126/sciadv.adz7406

This PDF file includes:

Supplementary Text S1 to S5
Figs. S1 to S9

1. Flash differential scanning calorimetry measurement

1.1. Reproducibility of the measurements

To evaluate the reproducibility of the flash differential scanning calorimetry (FDSC) measurements, we conducted at least two independent measurements for each sample. As shown in **Fig. S1**, three separate sample parts from one specimen (7 GPa and T_{comp}=643 K) were cut for independent FDSC measurements. The results show good reproducibility, with the endothermic peak areas and positions (deviation <1 K) essentially overlapping after mass normalization (see Methods in the main text).

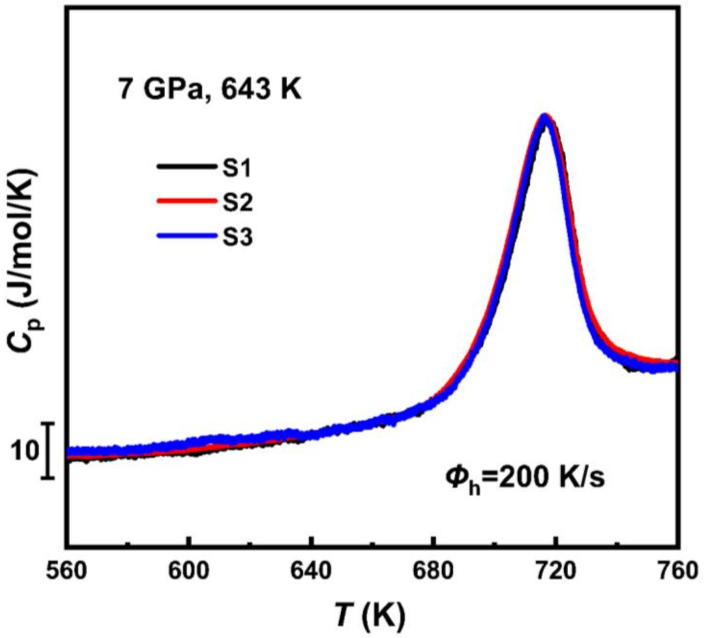

Fig. S1. Comparison of three independent FDSC measurements. Three different parts of the decompressed sample (P_{comp}=7 GPa and $T_{comp.}$=643 K) were cut out for FDSC testing, all performed at a heating rate of 200 K/s. The overlap between the different curves confirms the robustness of our data and thus of single shot FDSC approach.

1.2. Determination of the endothermic peak position

A consistent definition of the peak temperature for the endothermic peak was used for calculating the relaxation activation energy based on the Kissinger method. As shown in **Fig. S2**, we first performed a first scan (1st run) at a heating rate of Φ_h to the supercooled liquid plateau, then the sample was cooled at a rate of Φ_c (Φ_c = Φ_h) to 298 K, followed by a second scan to the supercooled liquid plateau (2nd run) at Φ_h. By

subtracting the curves of the 2nd run from the 1st run, the excess heat flow area was obtained, exhibiting a peak-like feature. The temperature corresponding to the maximum of the peak was defined as the characteristic peak temperature, T_p. This method was consistently applied across different heating and cooling rates to determine T_p.

As discussed in the literature (Ref. 67 in the main text), for α-relaxation, the Kissinger plot appears as a straight line at low heating rates, but when the heating rate exceeds a certain value, the data begins to deviate from the linear relationship of the Kissinger equation. This is considered to indicate that the glass transition cannot be simply viewed as a thermal activation process, especially in the high-temperature range. However, the Kissinger plot within a limited heating rate range (<1000 K/s) typically shows a linear relationship (as shown in **Fig. 2** in the main text) and provides a constant activation energy within a small temperature range. For the sub-T_g endothermic reaction (β-relaxation), however, over a wide heating rate range spanning four orders of magnitude, both the Kissinger equation and the Arrhenius equation provide excellent fits to the experimental results, and the activation energies estimated by both methods are comparable (Ref. 61 and 67 in the main text).

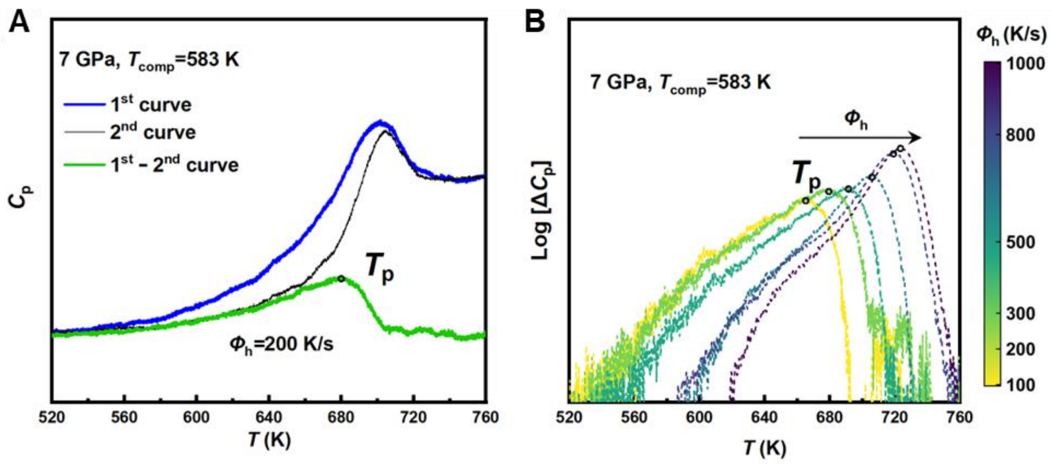

Fig. S2. Determination of the endothermic peak temperature, T_p at various heating rates. (A) 1st and 2nd runs performed at a heating rate of 200 K/s for a glass compressed at P_{comp}=7 GPa and T_{comp}=583 K. The second run corresponds to the subsequent reheating curve of the sample cooled from the supercooled liquid phase (cooling rate equals to the heating rate). The difference between the 1st and 2nd curve reveals a prominent characteristic peak, whose peak maximum position, T_p, is

determined by the temperature corresponding to the peak value of the curve (as marked by the black circle). **(B)** Characteristic peaks of the directly subtracted curves at different heating rates of 100-1000 K. The vertical axis is displayed on a logarithmic scale. The different T_p values and corresponding heating rates are reported in the Kissinger plot (**Fig. 2B** in the main text) and the slope of the Kissinger equation (see Methods in the main text) provides the activation energy of the relaxation process active during the compression.

1.3. Determination of the fictive temperature by area-matching method

To compare the thermodynamic states of the different samples, we calculated their respective fictive temperatures, T_f, using the area-matching method illustrated in **Fig. S3.** A smaller T_f indicates a more relaxed state, while a larger T_f means a less relaxed state. It is worth noting that, since all measurements are conducted on the state of the decompressed samples at 1atm, here T_f serves only as a parameter for comparing the thermodynamic states of the samples and does not represent the actual vitrification temperature under high pressure.

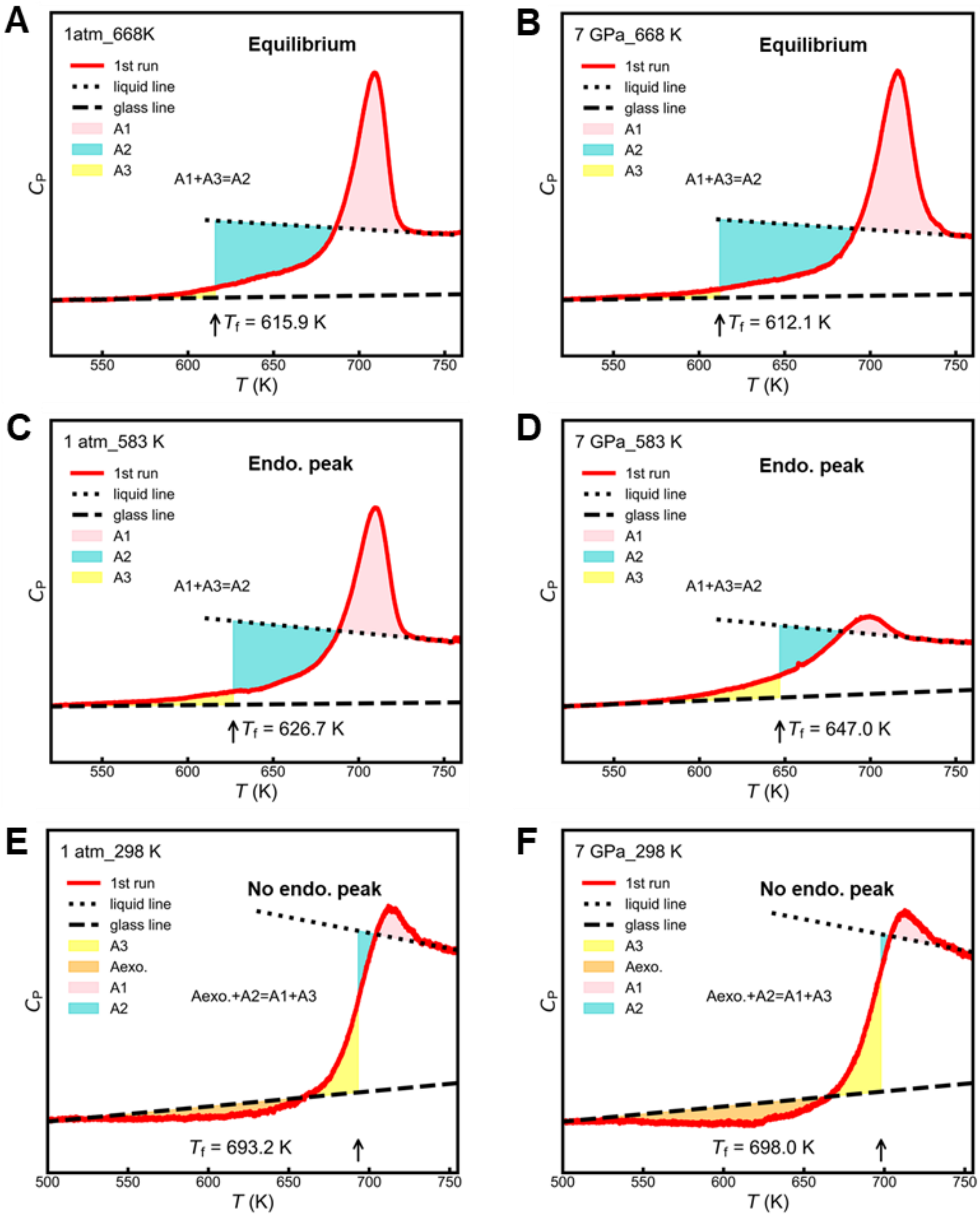

Fig. S3. Estimation of T_f for samples compressed and annealed at different temperatures. Illustration of T_f calculation using the area-matching method for samples in various states: **(A, B)** equilibrium state, **(C, D)** non-equilibrium state with an endothermic peak, and **(E, F)** non-equilibrium state with an exothermic peak.

1.4. Comparison of the area of endothermic peaks

The FDSC curves of glasses quenched from the supercooled liquid at 1 atm and 7 GPa are compared, highlighting differences in $T_{g,onset}$ (**Fig. 1B** in the main text). To facilitate a direct comparison of the endothermic peak shape and area, the temperature axis was normalized by the respective $T_{g,onset}$ of each sample. As shown in **Fig. S4**,

significant variations are observed in both the shape and area of the endothermic peaks. Compared to the 1 atm sample (T_a=643 K), the 7 GPa sample (T_{comp}=643 K) exhibits a larger endothermic peak area (indicating a lower T_f) and a broader peak (reflected by an increased ΔT). As discussed in the main text, the glass quenched from the supercooled liquid under high pressure reflect the "stronger" nature of corresponding dense liquid, while those prepared at ambient pressure are quenched from a more "fragile" liquid. This interpretation is further supported by the broadening of the glass transition overshoot peak observed in the FDSC scans. As shown in **Fig. S4**, the overshoot peak becomes broader in the high-pressure glass, indicating a wider glass transition interval. This is another hallmark of a stronger liquid, where the transition from the glass to liquid states occurs more gradually (Ref. 69 in the main text).

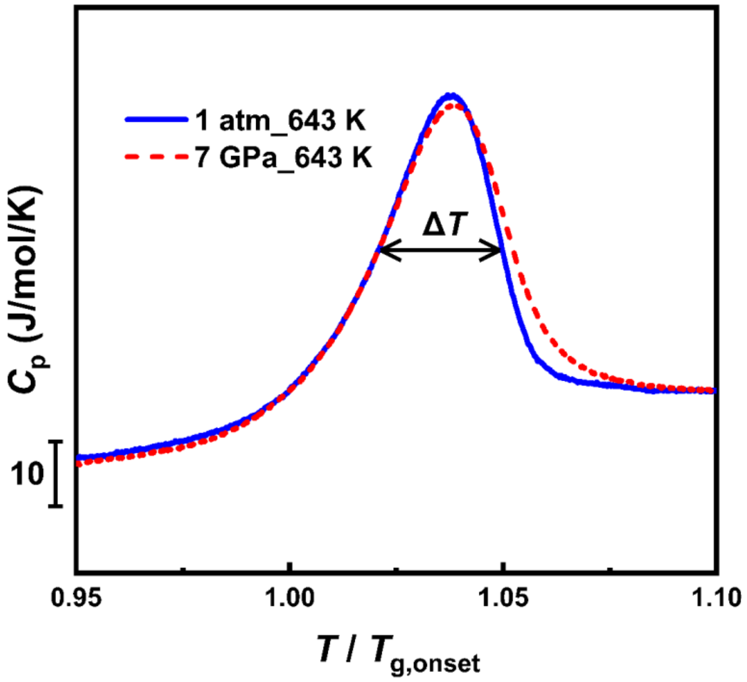

Fig. S4. Comparison of the endothermic peaks. FDSC curves of glasses quenched at 1 atm (T_a=643 K) and 7 GPa (T_{comp}=643 K), with temperatures normalized by $T_{g,onset}$ for easier comparison. ΔT represents the full width at half maximum of the endothermic peak.

2. Estimation of the dynamical slowdown

To estimate the extent of the α-relaxation slowdown under high pressure, we consider only a temperature shift of the Vogel-Fulcher-Tammann (VFT) function used

to describe the process at 1 atm. Since the activation energy of the α-relaxation varies very little with pressure (only about 4% lower than at 1 atm), our approach can still be considered reasonable and provides an upper limit to the pressure-induced increase in the α-structural relation time.

Based on the observed T_g increase at 7 GPa, a shift of 38 K toward higher temperatures is applied (**Fig. S5**). The 1 atm data are taken from Ref. 55 of the main text. On this basis, we estimate that the increase in T_g would correspond to a slowdown in the dynamics by a factor of approximately 1500 at the same absolute temperature under 7 GPa.

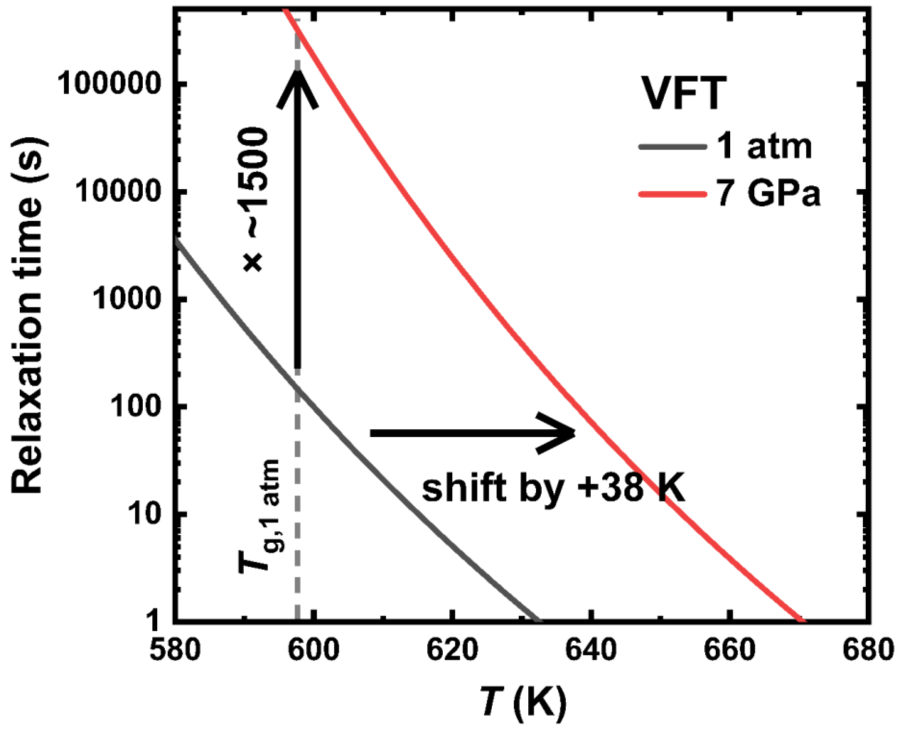

Fig. S5. Estimation of the dynamical slowdown. The relaxation times at 1 atm are taken from the results of stress relaxation reported in Ref. 55 of the main text. These data are fitted using the VFT equation: $\tau=\tau_0\exp[D^*T_0/(T-T_0)]$, with parameters D^*=19.8 (fixed) and T_0=383.9 K (black line). The VFT curve at 7 GPa (red line) is obtained by shifting the 1 atm VFT curve by 38 K toward higher temperatures.

3. Comparison between annealed and thermal compressed samples with the same degree of equilibration

As discussed in the main manuscript, the difference in T_f between thermal compressed samples and annealed glasses shown in **Fig. 3A** is a consequence of the different pressure dependencies of the α- and β-relaxation processes. From one side it arises partially from the slowdown of α-relaxation and the consequent pressure-induced increase in T_g, while on the other side, it depends on the irreversible structural rearrangements induced by pressure. To highlight the second factor, in **Fig. 3B, 3C** and **3D** we compare glasses with the same degree of equilibration. For this purpose, we select 3 different couples of glasses with the same normalized temperature as shown by the squares in **Fig. S6,** which are at ~0.87 $T/T_{g,P}$, ~0.95 $T/T_{g,P}$ and ~1.10 $T/T_{g,P}$. In this way, the selected couple of glasses have the same distance to equilibrium, i.e., the same $T/T_{g,P}$.

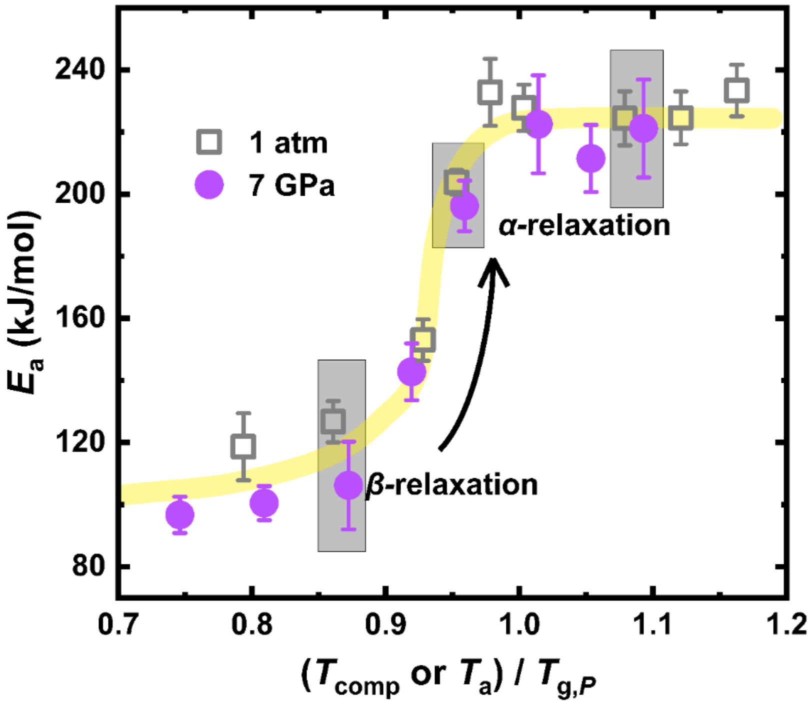

Fig. S6. Comparison of FDSC curves for glasses compressed at 7 GPa and annealed at 1 atm at comparable degree of equilibration (same reduced $T/T_{g,P}$) value. Evolution of the activation

energy E_a as a function of T_{comp} or T_a rescaled by $T_{g,P}$. The black squares indicate three sets of glasses with the same degree of equilibration at the two pressures that can be used for the comparison.

4. PDF comparison between the 1 atm and the compressed (T_{comp}=583 K) samples

As discussed in the main text, the glass compressed at 583 K does not show a significant change in bulk density compared to the sample quenched at ambient pressure. As shown in the **Fig. S7**, the positions of different coordination shells remain essentially unchanged relative to those of the 1 atm sample. In contrast, the observed structural rearrangements are likely due to a mechanically induced re-distribution of Zr–Zr and Cu–Zr atomic bonds at the level of short-range order. This interpretation is supported by the increased intensity of both the sub-peak (associated with Cu–Zr pairs) and the main peak (associated with Zr–Zr pairs) in the first coordination shell, as observed in comparison with the 1 atm sample.

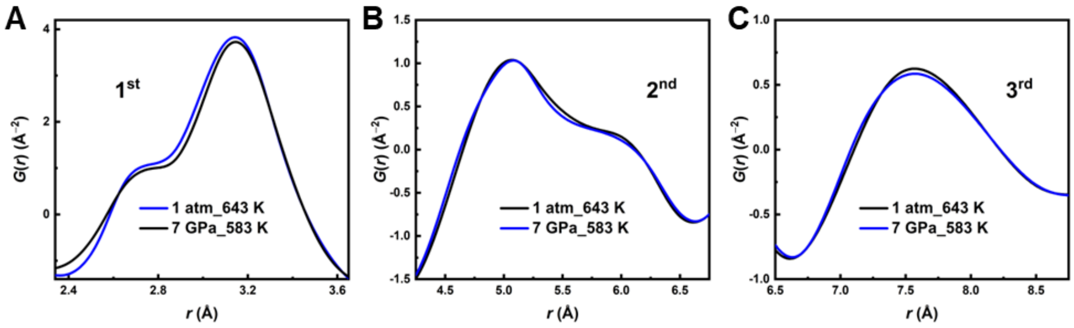

Fig. S7. Comparison of $G(r)$ between the 1 atm quenched sample and the compressed sample (T_{comp}=583 K). Panels **(A)**, **(B)**, and **(C)** show the information of the first, second, and third coordination peaks, respectively, for the two samples.

5. Analysis of the XRD data: fitting of q_1 and FWHM

In order to obtain q_1 and the FWHM for the first peak of $I(q)$, we employed Gaussian function fitting on the data:

$$y=y_0+\frac{A}{w\sqrt{\frac{\pi}{4\times\log(2)}}}\times\exp(-4\times\log(2)\times(\frac{x-x_c}{w})^2),$$

where y represents data from the first peak of $I(q)$, x represents the q values, y_0 is the baseline value, A is the amplitude of the function, x_c is the center of the peak, q_1, and w is the FWHM of the peak.

Figure S8 illustrates the correlation between the fitting results and the selection range of the fitted data, y_{pin}. The y_{pin} is defined as the percentage of the upper part of the peak height. Based on the fitting results for each curve, when y_{pin}<60%, all curves exhibit fitting with R^2>0.9999, indicating an excellent match between the Gaussian function and the XRD data. Considering the need to reflect as much information from the $I(q)$ peaks as possible, y_{pin} should not be too small. Therefore, in the main text, we used a y_{pin} value of 50% for fitting the data.

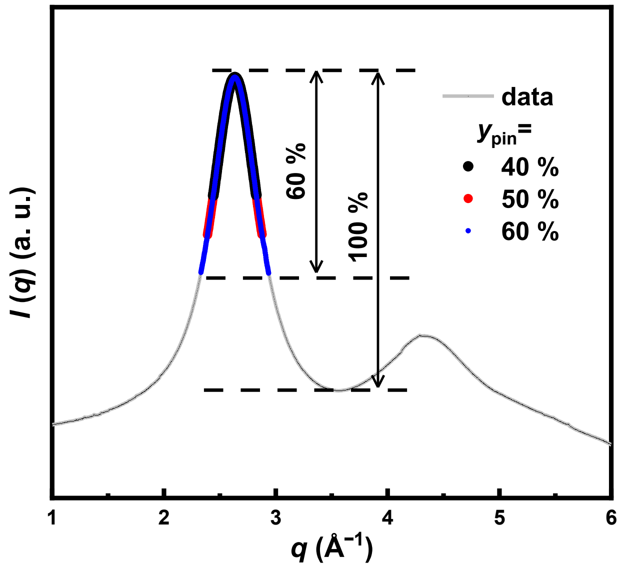

Fig. S8. Fitting approach of the XRD pattern. To define the fitting range of the Gaussian function, we characterized the normalized height, which is the difference of the maximum of the first peak to the minimum between the first and the second peak. y_{pin} represents the fitting range proportion of the normalized height. The black, red and blue points represent the data range for fitting with different y_{pin} values of 40%, 50%, and 60%.

Figure S9 Shows fits for y_{pin}=40%, 50%, and 60%. It is demonstrated that the

selected y_{pin} affects quantitatively the fitting value of the FWHM but does not alter its evolving trend, whereas values of q_1 remain almost unaffected.

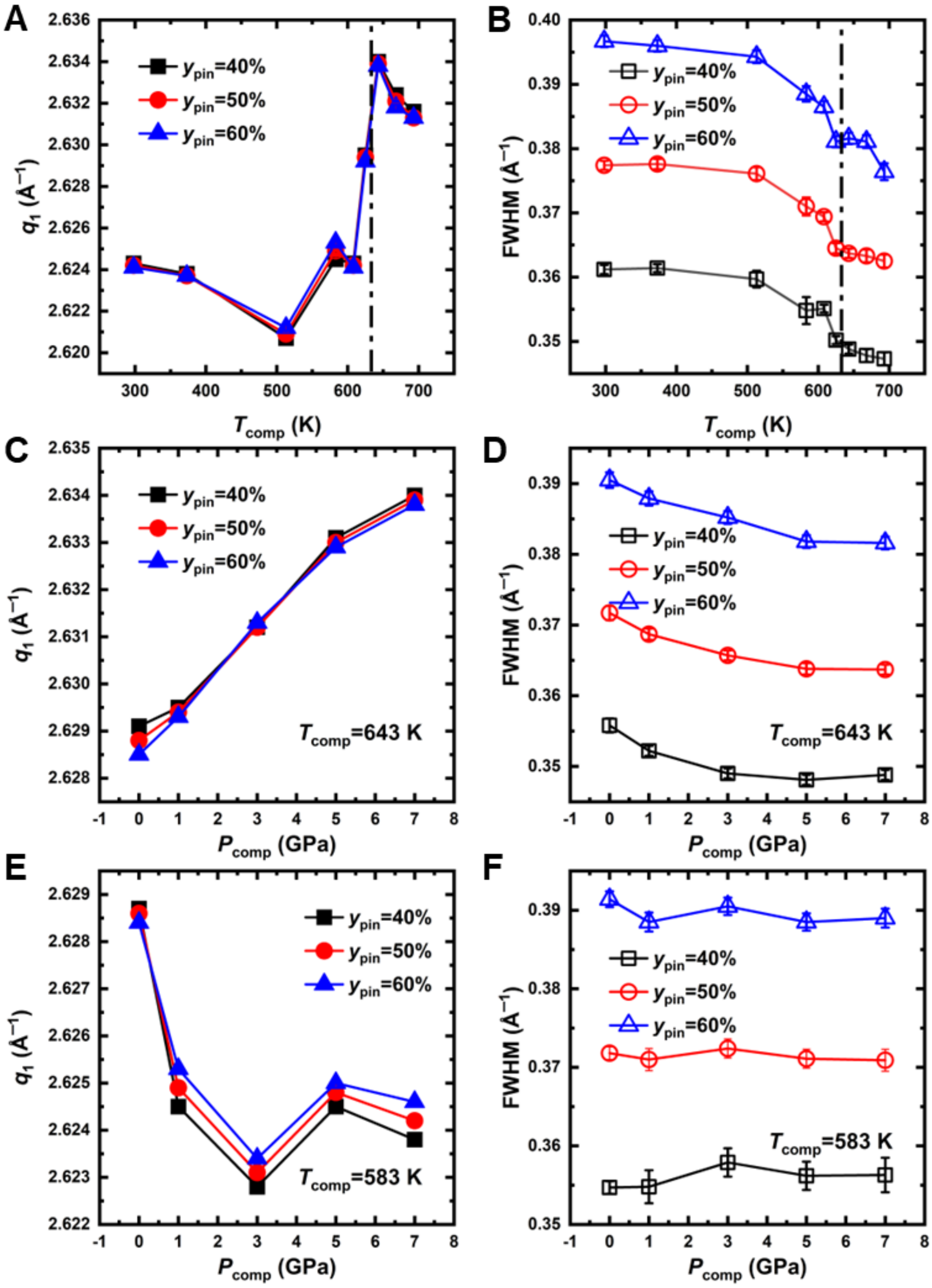

Fig. S9. (A, B) The q_1 and FWHM changes with T_{comp} as P_{comp}=7 GPa. **(C, D)** The q_1 and FWHM changes with P_{comp} as T_{comp}=643 K. **(E, F)** The q_1 and FWHM changes with P_{comp} as T_{comp}=583 K. The fits are derived from the data as y_{pin}=40%, 50% and 60%. The black dashed line in **(A, B)** corresponds to T_g at 7 GPa.
